# The hypomethylating agent Decitabine causes a paradoxical increase in 5-hydroxymethylcytosine in human leukemia cells

**DOI:** 10.1038/srep09281

**Published:** 2015-04-22

**Authors:** Basudev Chowdhury, Andrew McGovern, Yi Cui, Samrat Roy Choudhury, Il-Hoon Cho, Bruce Cooper, Timothy Chevassut, Amy C. Lossie, Joseph Irudayaraj

**Affiliations:** 1 Department of Biological Sciences, Purdue University, West Lafayette 47907, IN; 2 Bindley Biosciences Center, Discovery Park, Purdue University, West Lafayette 47907, IN; 3 Department of Healthcare Management and Policy, University of Surrey, Guildford, GY2 7XH, UK; 4 Brighton and Sussex Medical School, Falmer, Brighton, East Sussex, BN1 9PS, UK; 5 Department of Agricultural and Biological Engineering, Purdue University, West Lafayette, IN 47907; 6 Department of Animal Sciences, Purdue University, West Lafayette, IN 47907

## Abstract

The USFDA approved “epigenetic drug”, Decitabine, exerts its effect by hypomethylating DNA, demonstrating the pivotal role aberrant genome-wide DNA methylation patterns play in cancer ontology. Using sensitive technologies in a cellular model of Acute Myeloid Leukemia, we demonstrate that while Decitabine reduces the global levels of 5-methylcytosine (5mC), it results in paradoxical increase of 5-hydroxymethylcytosine (5hmC), 5-formylcytosine (5fC) and 5-carboxylcytosine (5caC) levels. Hitherto, the only biological mechanism known to generate 5hmC, 5fC and 5caC, involving oxidation of 5mC by members of Ten-Eleven-Translocation (TET) dioxygenase family, was not observed to undergo any alteration during DAC treatment. Using a multi-compartmental model of DNA methylation, we show that partial selectivity of TET enzymes for hemi-methylated CpG dinucleotides could lead to such alterations in 5hmC content. Furthermore, we investigated the binding of TET1-catalytic domain (CD)-GFP to DNA by Fluorescent Correlation Spectroscopy in live cells and detected the gradual increase of the DNA bound fraction of TET1-CD-GFP after treatment with Decitabine. Our study provides novel insights on the therapeutic activity of DAC in the backdrop of the newly discovered derivatives of 5mC and suggests that 5hmC has the potential to serve as a biomarker for monitoring the clinical success of patients receiving DAC.

Aberrant DNA methylation, such as hypermethylation of tumor suppressor genes, is a hallmark of cancer and a testament to the role of epigenetics in oncogenesis^1^,^2^,^3^. Epigenetic alterations on genes that regulate the differentiation of hematopoietic stem cells (HSCs) into matured blood cells, clinically christened hematopoiesis, are frequently observed in myeloid malignancies^4^,^5^,^6^,^7^,^8^. The recent discovery of the 5mC derivative, 5hmC^9^,^10^, in addition to its sub-derivatives, 5fC and 5caC^11^ (Figure 1a), has produced new players that could participate in epigenetic regulation of transcription^10^,^12^,^13^,^14^,^15^. The USFDA approved hypomethylating agent, Decitabine (5-aza-2′-deoxyctidine; Dacogen; DAC), removes 5mC marks through depletion of the maintenance methyltransferase DNMT1 in the cell. Although intuitively one would assume that DAC would induce demethylation randomly, data from colon cancer (HCT116) and HL-60 cell lines suggest that some loci may be protected from DAC-induced demethylation^16^,^17^.

During the semi-conservative DNA replication, DNMT1 is primarily responsible for transmitting the fidelity of cytosine methylation to the daughter cells^16^,^18^. Fully methylated CpG dinucleotides (5mC/5mC) are transiently transformed into hemi-methylated CpGs (5mC/C) that are recognized by DNMT1 and converted to fully methylated dinucleotides, thereby restoring the parental DNA methylation pattern (5mC/5mC)^19^. DNMT3A and 3B are traditionally recognized for their role as *de novo* DNA methyltransferases during early development and differentiation, although recently it has been proposed that DNMT3A and 3B may be involved in the DNMT1-mediated methylation process^19^. However, the only known mechanism facilitating the conversion of 5mC into 5hmC is mediated by members of the ten-eleven-translocation family of dioxygenase enzymes (TET 1, 2 and 3) via an Fe(II) and α-ketoglutarate (α-KG)-dependent oxidation reaction^10^. Loss-of-functional mutations in TET2 occur frequently in Myelodysplastic Syndromes (MDS) and Acute Myeloid Leukemia (AML)^6^,^7^,^20^, and are mutually exclusive of mutations in IDH1/2, whose aberrant product, 2-hydroxyglutarate, inhibits TET-mediated conversion of 5mC to 5hmC^21^. Mutations in DNMT3A are found in 22% of *de novo* AML cases^7^ where they sometimes are accompanied by mutations in either TET2 or IDH1/2, confirming the role of DNA methylation in normal hematopoiesis and leukemogenesis.

Although the precise molecular mechanism of DAC action is not completely understood, DAC is a structural analogue of cytosine and can easily be incorporated as its substitute in DNA during replication^22^. DNMT1 recognizes these Decitabine-Guanine dinucleotides as a natural substrate and initiates a methyltransferase reaction, but is trapped in the process^23^. This leads to depletion of DNMT1 from the cell, loss of maintenance methylation and ‘passive demethylation’ of genomic DNA following cell division^16^,^17^,^18^. The precise mode of maintaining 5hmC patterns during DNA replication is not known. Although TET proteins have been demonstrated to be capable of catalyzing both hemi-methylated and fully-methylated CpGs^10^, it is not clear if the TET proteins display a selective preference for hemi-methylated or fully-methylated CpG dinucleotides in cells.

In our present effort, we have studied the effects of DAC on the downstream derivatives of 5mC. Our study suggests a possible mechanism of action of DAC on 5mC derivatives, which could further our understanding of the effect of hypomethylating drugs and epigenetic therapies. To shed light on our observations, we employed a multi-compartmental model to mathematically interpret the DNA methylome changes and the underlying activities of TETs upon DAC treatment in human leukemia cells. We have also presented evidence in favor of our model, using advanced fluorescence microscopy and spectroscopy that has the ability to capture real-time single-molecule dynamics of TET proteins in living cells.

## Results

### Quantification of 5mC and 5hmC in HL-60 cells upon DAC treatment

**Figure 1:**
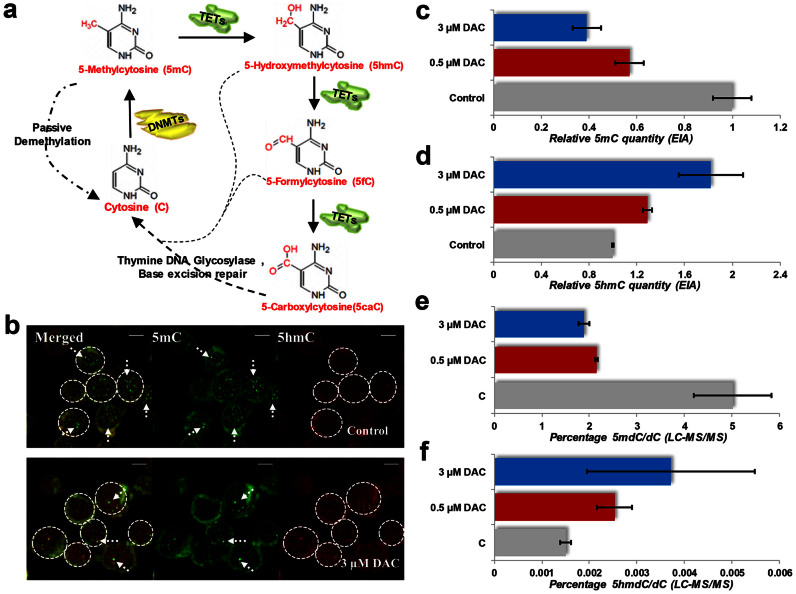
The effect of DAC on 5mC and 5hmC in HL-60 cells. (a) The scheme of mammalian ‘active demethylation’ pathway. (b) Immunocytochemistry for 5mC (green channel) and 5hmC (red channel) performed on untreated and 3 μM DAC treated HL-60 cells. The scale bar denotes 5 μm. (c & d) Global levels of 5mC and 5hmC by EIA respectively in untreated, 0.5 μM and 3 μM DAC treated HL-60 cells. The limit of detection of 5mC was 5 pg/100 ng of added DNA, while that for 5hmC was 2 pg/200 ng of added DNA. (e & f) LC-MS/MS quantitation of levels of 5mC and 5hmC in terms of ratios of 5-methyl-2′-deoxycytidine (5mdC) or 5-hydroxymethyl-2′-deoxycytidine (5hmdC) to those of deoxycytidine (dC) respectively in untreated, 0.5 μM and 3 μM DAC treated HL-60 cells. Limits of Detection (LOD) of 5mdC and 5hmdC were 0.09 and 0.11 fmol respectively.

Initially, we sought to determine the changes in the patterns of methyl-CpG-binding domain proteins (MBDs) in response to DAC, which led us to the unexpected observation that the 5hmC content in HL-60 cells increased in response to DAC. HL-60 cells, a well-studied acute myeloid leukemia cell line^24^,^25^, were treated with 0.5 μM and 3 μM DAC for 40 hours. The concentration of the drug used in our study closely resembles the range of maximum concentrations (*C_max_*) of DAC observed in human plasma at clinically administered dosages^26^,^27^. In untreated HL-60 cells, 5mC is localized at specific loci whereas 5hmC appears to be more dispersed throughout the nucleus. The reduction in the number of 5mC foci and appearance of more 5hmC foci upon DAC treatment is also visualized by immunofluorescence images (Figure 1b). Global analysis of DNA methylation by Enzyme-based Immunoassay (EIA) conceptualized in our lab^28^, revealed reduction in the levels of 5mC (Figure 1c) along with an increase in the levels 5hmC, following treatment (Figure 1d). In addition to the EIA quantification, liquid chromatography-tandem mass spectrometry (LC-MS/MS) analysis was used to further validate our observation of a decrease of 5mC and increase of 5hmC upon DAC treatment. We determined that the 5mdC/dC percentage in control, 0.5 μM DAC-treated and 3 μM DAC-treated HL-60 cells are 5.02%, 2.15% and 1.88% respectively (Figure 1e). By contrast, an increasing trend of the percentage of 5hmdC/dC was noted: from 0.0015% in control cells to 0.0025% in 0.5 μM DAC-treated cells, and to 0.0037% in 3 μM DAC-treated cells (Figure 1f). Recently it has been reported that, in addition to 5hmC, the TET proteins also generate 5fC and 5caC^11^. Interestingly, we observed a relative increase in the levels of 5fC and 5caC in HL-60 cells treated with DAC compared to control cells (Supplementary Figures 1a, b & c).

### Assessment of DNMTs and TETs

**Figure 2:**
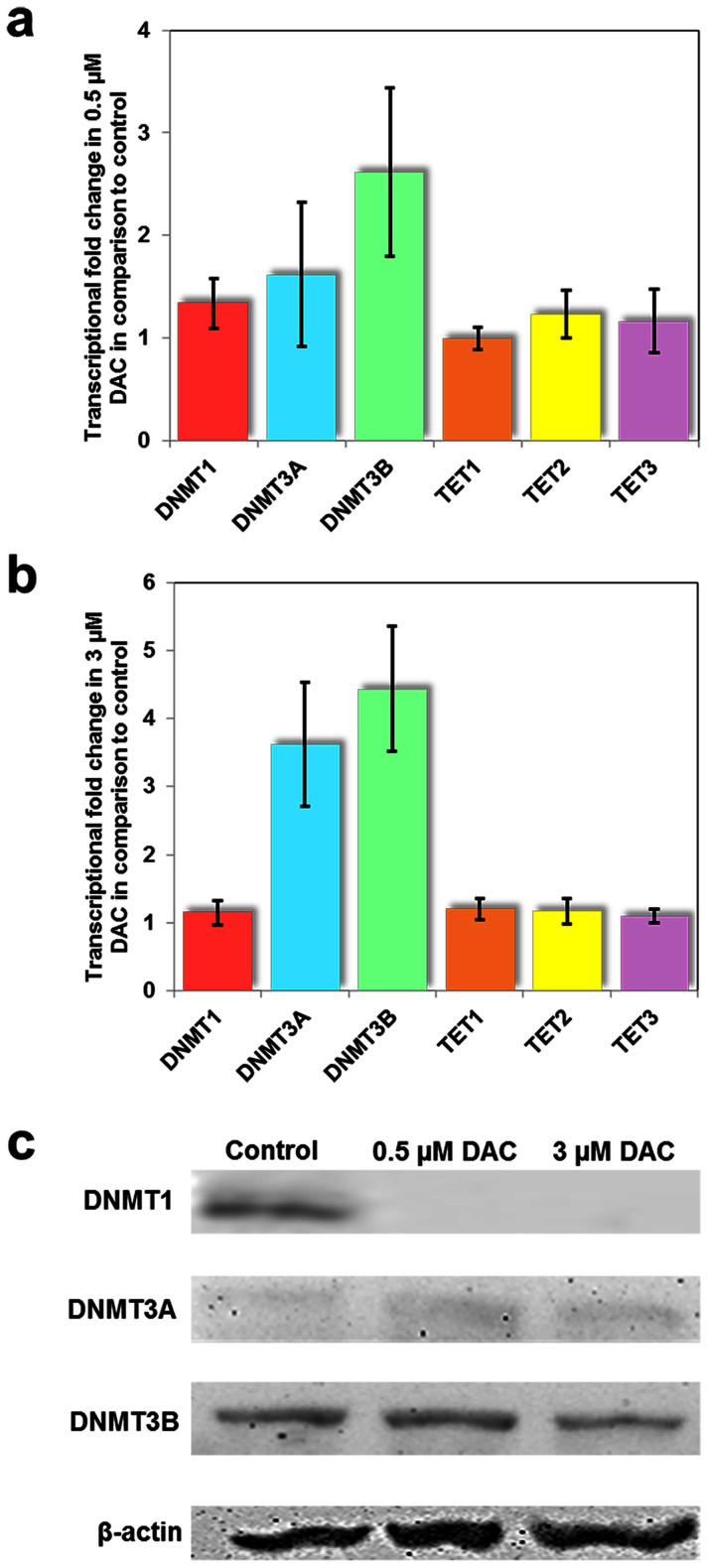
Molecular profiling of changes occurring during DAC treatment. (a & b) The transcriptional changes, measured by qPCR, of in DNMT1, DNMT3A, DNMT3B, TET1, TET2 and TET3 in 0.5 μM and 3 μM DAC treated HL-60 cells, respectively, compared to their levels in untreated HL-60 cells. All values have been normalized with GAPDH/β-actin (c) Western Blot analysis to understand the precise effect on DNMTs. β-actin is shown as a protein loading control.

To elucidate the mechanism of DAC-induced methylation changes, we measured the effect of DAC on the expression of DNMT and TET family members. While no significant changes in DNMT1, TET1, TET2 and TET3 transcript levels were observed, the steady state levels of DNMT3A and DNMT3B increased by 3.62 fold and 4.42 fold, (P < 0.05, two-tailed t-test respectively (Figures 2a & b). In contrast, at the protein level, we detected a near-complete reduction of DNMT1 (as expected), while DNMT3A and DNMT3B protein levels were unchanged (Figure 2c).

### Multi-compartmental modeling of TETs in DAC treated cells

**Figure 3:**
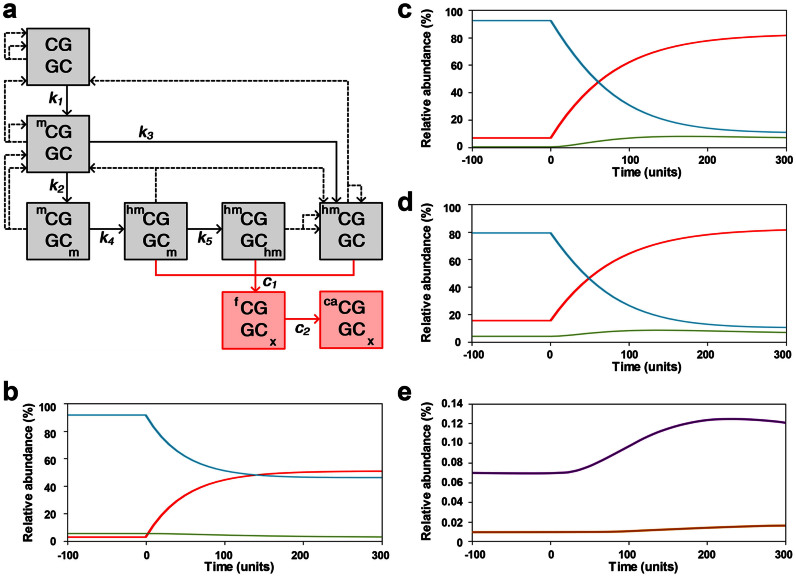
Mathematical simulation using a multi-compartmental model. (a) A schematic representation of the original six compartmental model components (black) and the extended model components (red). Solid lines indicate epigenetic modification by enzymes. Broken lines indicate the effects of cell division. The effects of DAC treatment (at time = 0) *in machina* on the relative abundance of unmethylated (red), methylated (blue), and hydroxymethylated (green) CpGs (b) in cells with non-selective TET; (c) in cells with TET proteins which are intrinsically fully selective for hemi-methylated CpG dinucleotides; (d) in cells with TET partially selective (6-fold) for hemi-methylated CpG dinucleotides; (e) and in cells with TET partially selective for hemi-methylated CpG dinucleotides demonstrating the effect on abundance of 5fC (purple) and 5caC (brown).

We modified a recent mathematical model (Figure 3a)^29^ to study the dynamics of methylation and demethylation and applied it to analyze the effect of DAC *in machina* and found that elimination of DNMT1 activity results in an increase of hemi-methylated CpG dinucleotides and loss of fully methylated CpG dinucleotides. The net effect of this change is a reduction in overall methylation levels and a corresponding reduction in 5hmC levels (Figure 3b). This global reduction of 5hmC is in contrast to our experimental data. However, an assumption of this mathematical analysis is that the TET proteins have equal affinity for both hemi-methylated and fully methylated CpG dinucleotides^29^. We therefore proposed that selective activity of TET for hemi-methylated dinucleotides (which have increased abundance after DAC treatment) could explain the apparent paradoxical increase of 5hmC, and extended our models to investigate this effect. Further, from our mathematical models, when TET was defined as being fully selective for hemi-methylated dinucleotides, cells treated *in machina* with DAC were found to have a dramatic increase in 5hmC levels (Figure 3c). However the pretreatment abundance of 5hmC in this system was substantially lower than experimentally reported levels^10^, suggesting that TET is unlikely to be fully selective for hemi-methylated DNA. In models where TET was defined as being partially selective for hemi-methylated dinucleotides, 5hmC levels were maintained in untreated cells yet increased in cells treated with DAC (Figure 3d). Alteration of the relative activity of TET on hemi-hydroxymethylated/hemi-methylated (5hmC/5mC) CpG dinucleotides had minimal effect on the overall levels of methylation or hydroxymethylation, primarily due to the low relative abundance of these dinucleotides. We further extended our model to show preliminary evidence of the effect of DAC on downstream derivatives of 5hmC (Figure 3a), namely 5fC and 5caC^11^, and found that an increased abundance of these minority nucleotides was also observed after DAC treatment (Figure 3e), consistent with our experimental data (Supplementary Figure 1).

### Single-molecule dynamics of TET1-CD-GFP after DAC treatment by Fluorescent Correlation Spectroscopy (FCS)

**Figure 4:**
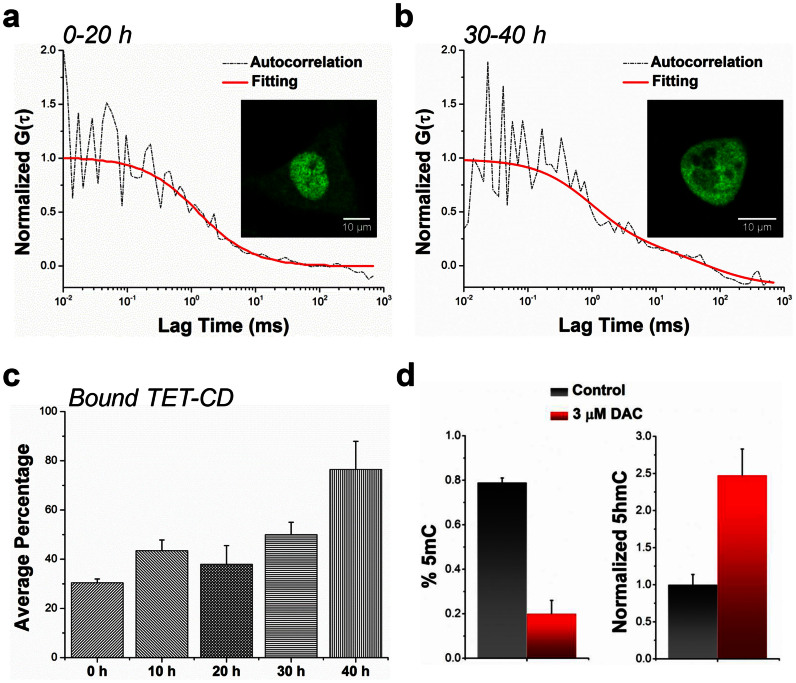
FCS measurement of TET-CD dynamics in MCF7 cells upon DAC treatment. (a) Representative autocorrelation function of TET-CD-GFP protein dynamics in early stage of DAC treatment. (b) Representative autocorrelation function of TET-CD-GFP protein dynamics in late stage of DAC treatment. (c) The percentage of bound TET-CD-GFP was calculated at different time points (n > 40 measurements for each point). (d) The corresponding changes of 5mC and 5hmC were determined after 40 hours of DAC treatment.

In order to experimentally support our modeling hypothesis, we constructed a GFP tagged TET1-CD (the domain shared by all other TET proteins) to inspect its DNA binding properties upon DAC incorporation. Following the molecular cloning and transfection of TET1-CD-GFP, we conducted FCS in MCF7 cells treated with DAC at different time points (Figures 4a & b). In the nucleus of untreated control cells, with an average expression of TET1-CD-GFP at ~200 nM, the diffusion times for free and bound TET1-CD-GFP are 1.79 ± 0.23 ms and 24.84 ± 5,74 ms, respectively. Notably, the free proteins constitute the predominant group – a percentage of ~73% (Supplementary Figure 2). With a gradual incorportation of DAC to generate hemi-methylated dyad along with DNA replication, a significant increase in the bound form of TET1-CD-GFP was noted from 30 hours. At 40 hours, the bound TET1-CD-GFP became the major diffusing form, accounting for a percentage of ~76% (Figure 4c). This result suggests that even although DAC treatment did not alter the amount of TET proteins, the DNA bound fraction of TET increased significantly while the free TET proteins decreasd simultaneously after DAC treatment, which in turn may cause an increase in 5hmC (Figure 4d).

## Discussion

The “epigenetic drug”, Decitabine has a dose-dependent mode of action^30^. While at higher doses, it is a potent cytotoxic agent; at chemically administered lower doses, it is an effective anti-tumour agent^30^,^31^,^32^. Elaborate studies involving Next-Generation Sequencing of the reduction in global levels of 5mC in leukemia cells treated with DAC indicate non-random patterns of demethylation^16^,^17^,^18^, with an unexplained up-regulation of genes involved in differentiation^16^ and those involved in tumor suppression (P15/CDKN2B^33^, TP73^33^, Cadherin1^33^,^34^, MLH1^35^ and P16/CDKN2A^36^). However, the discovery of downstream derivatives of 5mC, namely, 5hmC, 5fC and 5caC compelled us to re-investigate the effect of DAC on these novel epigenetic marks.

The low dosage of DAC administered in our study led to a mild inhibition in the rate of cellular proliferation (Supplementary Figure 3); minor differences in the cell cycle and apoptotic profile (Supplementary Figure 4a & b), and reduction in levels of 5mC (Figure 1c & e) compared to control, consistent with previous reports^17^,^30^,^37^. On the other hand, we report the very first observation of an increase of 5hmC content in DAC treated HL-60 cells (Figure 1d & f). While trapping of DNMT1 proteins followed by proteasomal degradation in the presence of DAC has been demonstrated experimentally^18^, there is controversy regarding the effect of DAC on DNMT3A and DNMT3B^38^,^39^,^40^. We were perplexed by the incongruence between steady-state mRNA and protein levels of DNMT3A and DNMT3B following DAC exposure. However, a transcription independent decrease in DNMT3A protein levels has been reported in hypomethylated cells with impaired DNMT1 activity^41^, which supports our findings. We found no significant alterations in TET1, TET2 and TET3 transcript levels following DAC exposure.

A number of mathematical models have attempted to capture the complex mechanisms involved in methylation^42^,^43^ and more recently, have included the ‘active demethylation’ effects of the TET proteins (Figure 3a)^29^. Our *in machina* models suggest that the apparent paradoxical increase in 5hmC levels following DAC treatment of HL-60 cells can be explained if TET enzymes preferentially act at hemi-methylated rather than fully methylated CpG dinucleotides. It remains unclear whether this is due to DAC or an intrinsic property of the TET proteins. Due to the limited availability of tools to study the dynamics of CpG dinucleotides bearing derivatives of the methylation-demethylation cycle, gathering evidence in support of the conclusion of the mathematical modeling was a daunting task. However, we exploited the recent advances in the field of FCS technique to assess the real-time account of molecular events resulting during DAC treatment. Biophysically, TET proteins are considered to have distinct diffusion properties on the basis of whether it is found freely in the nucleus or bound to the DNA (Supplementary Figure 2). The confocal alignment of FCS facilitates collection of fluorescence events in a volume of less than 1 femtoliter at a low laser power (1 μW) at physiological conditions, giving it a distinctive advantage over other methods^44^,^45^,^46^,^47^,^48^,^49^.

TET mediated conversion of methylated and hemi-methylated oligonucleotides into their hydroxymethylated and hemi-hydroxymethylated forms, respectively, has been previously reported^10^, but the relative affinities of TET proteins for fully methylated and hemi-methylated CpGs need to be experimentally determined. In this regard, we have for the first time, studied the dynamics of TET1-CD-GFP by FCS in live cells after DAC treatment and have reported the significant increase in the DNA-bound fraction of TET upon DAC treatment. Similar to DNMTs^41^,^50^,^51^, in the nucleus, the DNA bound fraction of TET proteins can be correlated with enzymatic activity^52^. Thus, the significant increase of the DNA-bound fraction of TETs from 27% at 0 hour to 76% after 40 hours of DAC treatment, suggests significant alteration of TET dynamics and its consequent enzymatic activity in cells after administration of DAC.

**Figure 5:**
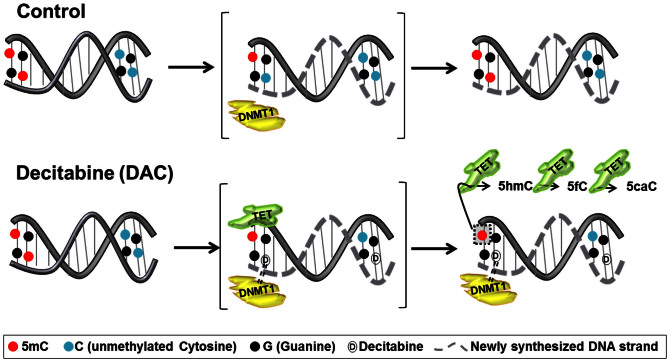
Our proposed model to explain the increase of 5hmC, 5fC and 5caC upon DAC treatment. The TET proteins appear to be partially selective for hemi-methylated CpGs, and in absence of DNMT1 (due to covalent binding with DAC), can convert the hemi-methylated CpGs into hemi-hydroxymethylated, hemi-formylated or hemi-carboxylated CpGs. Semi-conservative DNA replication in the presence of DAC may give rise to a condition where some methylated-CpGs incorporate DAC in place of Cytidine in the newly synthesized strand while the parent strand maintains the original 5mC mark (i.e. resulting in 5mC-G/G-DAC dinucleotide). In absence of DNMTs (DAC induced trapping and degradation) or knowledge of non-TET mediated active demethylation pathway in mammalian cells, it may be likely that the TETs can act on the 5mC of the parent strand, converting it to the other derivatives in the active demethylation pathway (5mC-G/G-DAC → 5hmC-G/G-DAC → 5fC-G/G-DAC → 5caC-G/G-DAC). Similarly, 5hmC or 5fC of the hydroxymethylated-CpGs and formylated-CpGs respectively incorporating DAC in the daughter strand could get converted to downstream derivatives of the pathway.

All of this taken together suggests that the increased recruitment of TETs on DNA after DAC treatment, possibly at hemi-methylated dyads, maybe one of the critical factors responsible for the increased abundance of 5hmC and the other derivatives of 5mC. Thus we hypothesize that, incorporation of DAC in the context of methylated CpG dinucleotides during semi-conservative DNA replication leads to trapping of DNMT1 and consequent conversion of the 5mC in such hemi-methylated CpGs to its downstream derivatives by TETs (Figure 5). Our findings suggests that the advent of TET proteins following DNMT1 trapping and degradation, can be due to their enzymatic preference for hemi-methylated CpGs, which are increased in abundance following DAC administration. Following these lines of experiments, we further validated the similar changes of 5mC and 5hmC in TK6 and MCF7 cells upon DAC treatment (Supplementary Figure 5).

Finally, we believe that our observations have important implications in the putative therapeutic mechanism of action of hypomethylating agents currently in clinical usage in MDS and AML. Hematological cancers are characterized by recurrent mutations in genes involved in the regulation of DNA methylation, notably TET2, IDH1/2 and DNMT3A. Our data suggest that in trying to understand the biological effects of DAC and related epigenetic therapies in certain cancers, we must consider the effects of the drug on the levels of 5mC and its downstream derivatives, and propose that 5hmC may serve as an useful biomarker to monitor the clinical success of patients administered with such epigenetic therapies.

## Methods

### Cell culture and DAC treatment

HL-60 cells (a kind gift of Dr. Ann Rundell, Purdue University) and TK6 cells were grown in RPMI medium (Life Technologies) and supplemented with 10% fetal bovine serum (Atlanta Biologicals Inc) and 1% Penicillin- Streptomycin (Life Technologies). MCF7 cells were routinely cultured with DMEM/F-12 based medium. The cells were incubated for 24 hours in media prior to treatment with 0.5, 1, 3 and 5 μM DAC for 40 hours (Sigma-Aldrich Corp). All experiments were replicated at least 3 times. Cell viability was calculated by Trypan blue exclusion staining (Supplementary Figure 3).

### DNA, RNA and protein extraction

Genomic DNA, total RNA and proteins were extracted from control and DAC treated HL-60 using standard protocols. Briefly, genomic DNA was extracted by DNeasy Blood &Tissue kit (Qiagen), total RNA extracted by RNeasy Mini Kit (Qiagen) and total proteins by RIPA buffer (Thermo Fisher Scientific) following manufacturer's instructions.

### Quantitative analysis of 5mC, 5hmC, 5fC and 5caC by Enzyme-Linked Immunoassay (EIA)

The quantitation of 5mC and its demethylation derivatives were performed by the EIA platform developed in our lab and as described previously^28^.

### Quantitative analysis of 5mC and 5hmC by LC-MS/MS

Genomic DNA was digested into constituent nucleosides using a nuclease mix (Zymo Research, Irvine, CA) and chromatographic separation of nucleosides was performed using an Agilent 1200 HPLC system with a Waters Atlantis T3 (2.1 × 150 mm, 3.5 μm) reversed-phase column, with a flow rate of 0.3 ml/min at ambient temperature. The mobile phase consisted of A (0.1% formic acid in water) and B (0.1% formic acid in acetonitrile), starting with 0% B for 2 minutes, with a linear gradient of 0–10% B from 2–8 minutes, with a linear gradient of 10–60% B from 8–10 minutes, a hold at 60%B from 10–12 minutes. Column re-equilibration was performed of 60–0% B from 12–13 minutes, with a 0% B hold from 13–23 minutes. Online mass spectrometry detection was performed using an Agilent 6460 triple quadrupole mass spectrometer, utilizing positive electrospray ionization mode. The deoxyribonucleosides were evaluated by Multiple Reaction Monitoring using the indicated mass transitions: 228.2 → 112.1 (dC), 242.2 → 126.1 (5mdC), 258.2 → 142.1 (5hmdC), 256.1 → 140.1 (5fdC) and 272.1 → 156.1 (5cadC). 5mdC, 5hmdC and dC were quantitated using calibration curves generated from authentic standards. The linear range and limits of detection are listed in Supplementary Table 1. Limits of Detections (LODs) of 5mdC and 5hmdC (0.09 and 0.11 fmol respectively) were comparable to the LODs obtained by the most sensitive LC-MS/MS method (devised by Chen et al^50^). 5fdC and 5cadC were computed on the basis of peak areas since authentic standards were not commercially available.

### Immunocytochemistry (ICC)

HL-60 cells were fixed in 4% paraformaldehyde for 15 minutes. The cells were then washed with cold PBS and smeared on poly-L lysine coated coverslip. The cells were then permeabilized for 15 minutes with cold PBS containing 0.4% Triton X-100. Permeabilized cells were then washed and denatured with 2 N HCl for 15 minutes, neutralized with 100 mM Tris-HCl (pH 8.5) for 10 min. The cells were then incubated for 1 hour with blocking buffer (10% goat serum, 3% bovine serum albumin in PBS containing 0.1% Triton X-100) before incubation with primary antibodies [For 5mC (Eurogentec) or 5hmC (Active Motif)] overnight at 4°C. After three consecutive 5-min washes with PBS, cells were incubated with secondary antibodies for 30 minutes. Cells were washed again three times with PBS and then mounted in fluorescent mounting medium (Vector Laboratories). Images were acquired using Nikon A1R multiphoton microscope and analyzed by Nikon software.

### Quantitative RT-PCR (qRT-PCR)

cDNA was generated from total RNA extracted from cells with Quantitect Reverse Transcription Kit (Qiagen). qRT-PCR reactions were performed on an ABI PRISM StepOnePlus Real-Time PCR system (Applied Biosystems) using SYBR Green reagent (Life Technologies) or Taqman dye (Life Technologies). cDNA levels of target genes were analyzed using comparative *C_t_* methods, where *C_t_* is the cycle threshold number, and normalized to GAPDH. PCR primers are listed in Supplementary Table 2. Taqman probes for TET1, 2, 3 were purchased from Life Technologies.

### Western blot

30 μg of cell lysates were analyzed on 8% Tris-Glycine polyacrylamide gels (Invitrogen) and transferred to PVDF membrane using BioRad mini transfer apparatus for 1.5 hour at 50 V. Membrane was blocked with 5% milk for one hour in room temperature and probed with Rabbit polyclonal anti-DNMT1 (Abcam, 1:1000), DNMT3A (Abcam, 1:500), DNMT3B (Abcam, 1:500), β-actin (Abcam, 1:500), Tet1 antibody (Abcam, 1:1000) or mouse monoclonal anti-TET2 (Active Motif 1:500) for overnight at 4°C. This was followed by washes in PBST (0.5% Tween 20) and incubation in HRP-conjugated secondary antibodies (Cal Biochem). Blots were developed using ECL reagent (GE Healthcare).

### Mathematical analysis

In the most recent mathematical model of DNA methylation^29^ the relative activities of the various proteins are represented by a series of constants (*k_1_, k_2_, k_3_, k_4_, and k_5_*). These constants characterize the flow between six compartments, each representing a discrete epigenetic CpG modification. In brief, *k_1_* represents the combined activity of DNMT3A and DNMT3B to generate hemi-methylated CpG dinucleotides, *k_2_* the activity of DNMT1 to generate fully methylated CpG dinucleotides, and *k_3_-k_5_* the activity of TET to perform hydroxymethylation on hemi-methylated, fully methylated, and hemi-hydroxymethylated and hemi-methylated (5hmC/5mC) CpG dinucleotides respectively. The complete model comprises a set of first order, partial differential equations which can be solved via numerical integration.

*In machina* treatment with DAC was modeled as elimination of DNMT1 activity i.e. as *k_2_* = 0. To test the hypothesis that selective activity of TET for hemi-methylated dinucleotides could explain the apparently paradoxical increase in hydroxymethylation the hydroxymethylation constants *k_3_* and *k_4_* were varied. Where TET was defined as being fully selective for hemi-methylated dinucleotides a value of *k_4_* = 0. In models where TET was defined as being partially selective for hemi-methylated dinucleotides, the relative values of the hydroxymethylation constants were varied such that *k_3_* > *k_4_*. When using a value of *k_1_* = 0.2 (consistent with previous models^29^,^43^) a value of *k_3_/k_4_* of 0.5 or greater was found to produce an increase in hydroxymethylation when DAC treatment was applied (although at the lower *k_3_/k_4_* values this increase was transient). We found that modification of *k_5_* had minimal effect on the model which is likely a result of the relatively small abundance of hemi-hydroxymethylated hemi-methylated (hmC/mC) dinucleotides. All models were analyzed using fourth order Runge-Kutta numerical integration.

In order to extend the model to incorporate the downstream products of TET activity 5fC and 5caC two additional partial differential equations were incorporated into the existing model. The first enables calculation of the change in abundance of 5fC (*x_7_*) in the cell population given the abundance of 5hmC/5mC (*x_4_*), 5hmC/5hmC (*x_5_*), and 5hmC/C (*x_6_*):
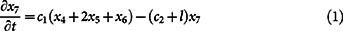
where *c_1_* is the conversion rate constant of 5hmC to 5fC, *c_2_* is the conversion rate constant of 5fC to 5caC, and *l* is the cell loss rate. The second equation enables calculation of the change in abundance of 5caC (*x_8_*) in the cell population given of the abundance of 5fC (*x_7_*):
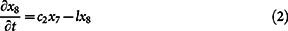
As the relative abundance of 5fC and 5caC is very small^11^ no changes were made to the existing system of equations assuming that movement of dinucleotides into and out of these two new model compartments will have a negligible impact on the far greater abundances of the other types of dinucleotide. Values of c1 = 0.00352 and c2 = 0.0288 were selected to give abundances of 1:65 and 1:430 for 5fC and 5caC respectively relative to 5hmC in the steady state, as reported for murine ES cells^11^. When *in machina* DAC treatment was applied to this extended model 5fC and 5caC abundances increased in tandem with 5hmC abundance when TET was selective for hemi-methylated dyads.

### TET1-CD-GFP construct and transfection

The fusion protein of TET1 catalytic domain (TET1-CD) and EGFP was generated by sequentially assembling the coding sequences of the desired proteins using standard restriction enzyme digest and ligation method. The inserts are incorporated into the pShooter™ mammalian expression vector (V821-20, Life Technologies). Prior to the incorporation of inserts, an adapter molecule was introduced in the multiple cloning sites (MCS) of the vector with *NcoI* and *PstI* flanking at the 5′ and 3′ end respectively. Briefly, the adapter molecule was generated by annealing the equimolar concentration of the primers UP: 5′-CATGGATCCGAGGCGCGCCGCTAGCGGTACCCTGCA-3′ and LP: 5′-GGGTACCGCTAGCGGCGCGCCTCGGATC-3′ at 50°C for 10 min. The adapter thus made was ligated to the vector after double digestion with *NcoI* and *PstI*. This adapter molecule introduced new restriction sites to the vector, including *BamHI, AscI, NheI, KpnI*, and *SfbI*. The source plasmids of TET1 (#49792 from Anjana Rao lab), and EGFP (#23027 from Andrea LeBlanc lab) were obtained from the Addgene plasmid repository (https://www.addgene.org). EGFP and catalytic domain of TET1-CD were PCR amplified with desired restriction sites flanking on either side, from the respective source plasmids. Suitable linker molecules were included to the primer sequences, where needed. The PCR reaction was carried out as specified by the manufacturer (CloneAmpHiFi PCR Premix; Clontech) for the template DNA concentration > 100 ng with 35 cycles of amplification. Details of the PCR primers have been summarized in Supplementary Table 3. Digested PCR amplicons were gel purified using the QIAEX II gel extraction kit (Qiagen). Purified vector and inserts thus made were ligated along with requisite amount of T4 ligase buffer and T4 DNA ligase (New England Biolabs) and kept at room temperature for 15 minutes. The ligated products were then transformed into the stellar™ competent cells (PT5056-2, Clontech) and plated out on Ampicillin containing LB agar plate. Suitable clones were propagated and the plasmids were extracted with QIAprep Spin Miniprep Kit (Qiagen). MCF7 cells for transfection were seeded on sterilized No.1 coverslips (VWR International) in a 12-well plate. After reaching 70% of cell confluence the medium was replaced with antibiotic-free, low-serum medium for another 12 hours. Then 400 ng of plasmid DNA was added to the culture with Lipofectamine® LTX transfection reagents (Life Technologies). Transfection efficacy was tested after 24 hours prior to the single-molecule measurements.

### FCS measurement of TET1-CD-GFP after DAC treatment

The intracellular dynamics of TET1-CD-GFP upon 3 μM DAC treatment was measured by FCS in a scanning confocal time-resolved system (Microtime200, Picoquant). A 465-nm picosecond pulsed diode laser was delivered through a 60× water immersion objective (N.A. = 1.2) to excite the GFP tag. The emitted fluorescence was collected by single photon avalanche photodiode detector (SPAD, PerkinElmer) and stored in time-correlated single-photon counting (TCSPC) module. The iteration of the autocorrelation function *G(τ)* for FCS is based on:
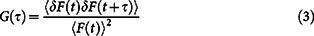
where *F* is the fluorescence intensity; *τ* is the time delay. To obtain the diffusion state of TET-CD-GFP, a 3D two-component diffusing model was applied:
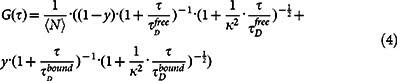
where *N* is the average number of molecules, *y* is the percentage of bound TET-CD-GFP, *τ_D_* is the diffusion time, and *κ* is the excitation profile.

## Supplementary Material

Supplementary Information Supplementary Information
