# Supplementary material for: The hypomethylating agent Decitabine causes a paradoxical increase in 5-hydroxymethylcytosine in human leukemia cells
Source: Sci Rep. 2015 Apr 22;5:9281. doi: 10.1038/srep09281 (PMC4894448; doi:10.1038/srep09281)
Supplement: Supplementary Information [file srep09281-s1.pdf]

SUPPLEMENTARY INFORMATION

The hypomethylating agent Decitabine causes a paradoxical increase in 5-hydroxymethylcytosine in human leukemia cells

*Basudev Chowdhury[1,2], Andrew McGovern[3,4], Yi Cui[2,5], Samrat Roy Choudhury[2,5], Il-Ihoon Cho[2,5], Bruce Cooper[2], Timothy Chevassut[4], Amy C. Lossie[2,6] and Joseph Irudayaraj[2,5]**

[1] Department of Biological Sciences, Purdue University, West Lafayette 47907,IN

[2] Bindley Biosciences Center, Discovery Park, Purdue University, West Lafayette 47907, IN

[3] Department of Healthcare Management and Policy, University of Surrey, Guildford, GY2 7XH, UK

[4] Brighton and Sussex Medical School, Falmer, Brighton, East Sussex, BN1 9PS, UK Department of

[5] Agricultural and Biological Engineering, Purdue University, West Lafayette 47907, IN

[6] Department of Animal Sciences, Purdue University, West Lafayette 47907, IN

*****Address for correspondence:

Joseph Irudayaraj, Professor

225 South University Street

Purdue University

West Lafayette, Indiana, 47907

Tel: 765-494-0388 Fax: 765-496-1115

Email: josephi@purdue.edu

SUPPLEMENTARY FIGURES AND TABLES

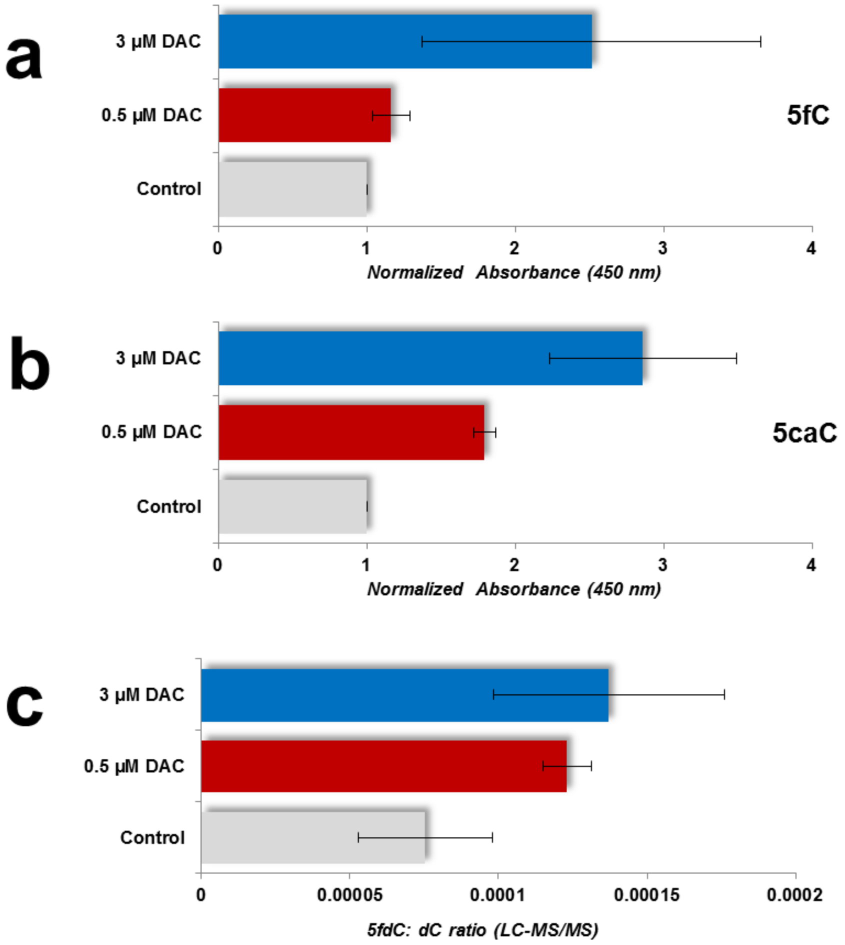

Supplementary Figure 1 *The effect of DAC on 5fC and 5caC in HL-60*. **(a & b)** Normalized values of global levels of 5fC and 5caC by EIA respectively in untreated, 0.5 µM and 3 µM DAC treated HL-60 cells. The limits of detection of 5fC and 5caC could not be precisely quantitated. The normalized O.D 450 nm readouts were thus normalized. **(c)** LC-MS/MS quantitation of levels of 5fC in terms of ratios of 5-formyl-2'-deoxycytidine (5fdC) to those of deoxycytidine (dC) in untreated, 0.5 µM and 3 µM DAC treated HL-60 cells. The levels of 5caC could not be ascertained by LC-MS/MS, probably due to its occurrence in extremely low amounts in the DNA.

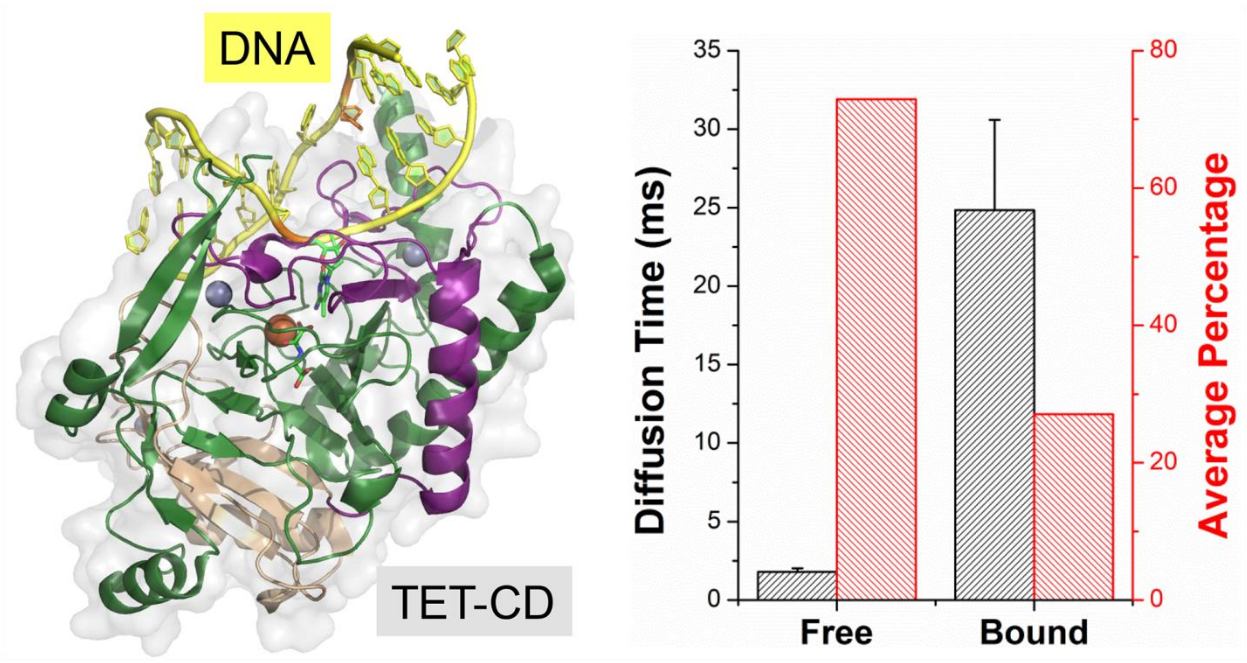

Supplementary Figure 2 *The association between TET-CD and DNA in control cells*. In the right panel, the binding or free-diffusing state of TET-CD was distinguished based on its characteristic diffusion time, and the percentage for each component can be calculated.

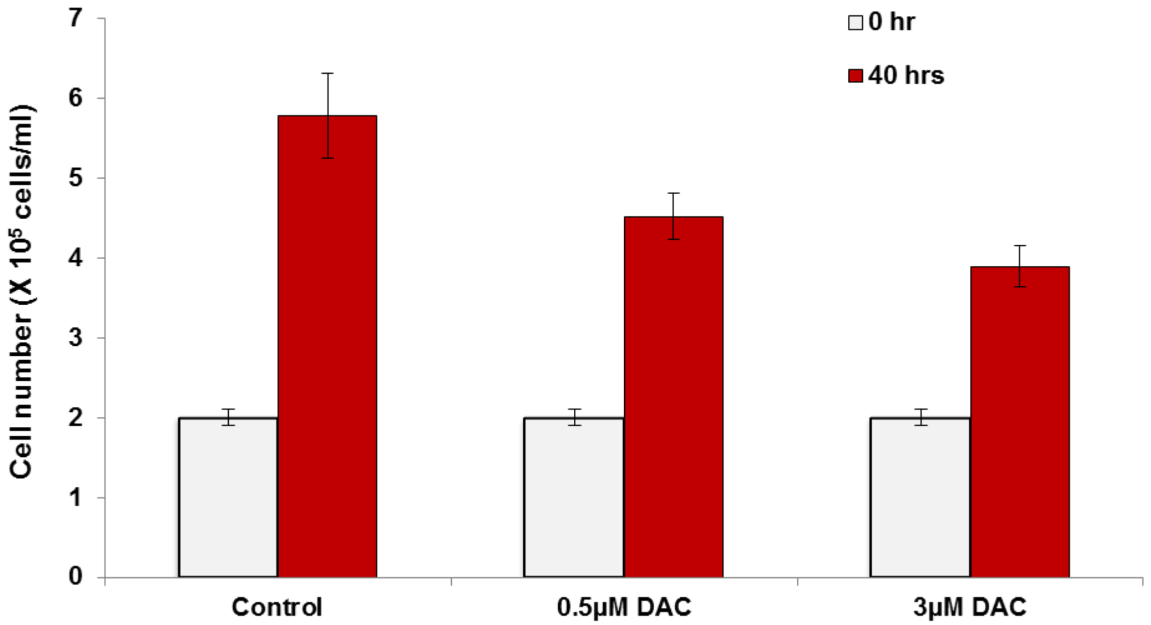

Supplementary Figure 3 *DAC affects cell viability in HL-60 cell line*. Cell viability of AML cell line (HL-60) was assessed after 40 hours of treatment with DAC, using direct cell counts with Trypan Blue Exclusion Assay. Standard deviation was determined from replicates of atleast 5 independent experiments. At 0 hour, the number of cells in every biological condition was identical ($2X10^5$ cells/ml).

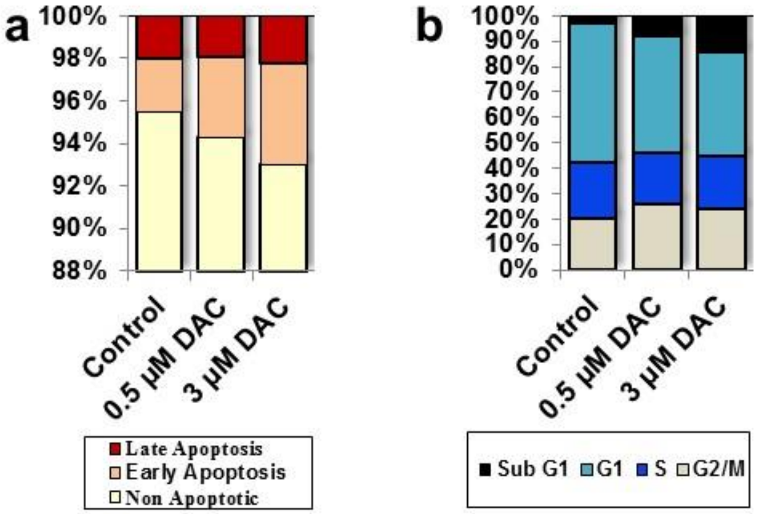

Supplementary Figure 4 *Apoptosis and cell cycle analysis* **(a)** DAC induces apoptosis in HL-60 cells. Apoptosis was detected with flow cytometry by positive staining for Annexin V (early apoptosis) and 7-AAD (late apoptosis). **(b)** Cell cycle analysis performed 40 hours after drug treatment. Cells were fixed in 70% ethanol, stained with Propidium Iodide (PI) after RNase treatment and quantified by flow cytometry for percentage of cells in sub-G1, G0/G1, S, and G2-M phases (normalized to 100%).

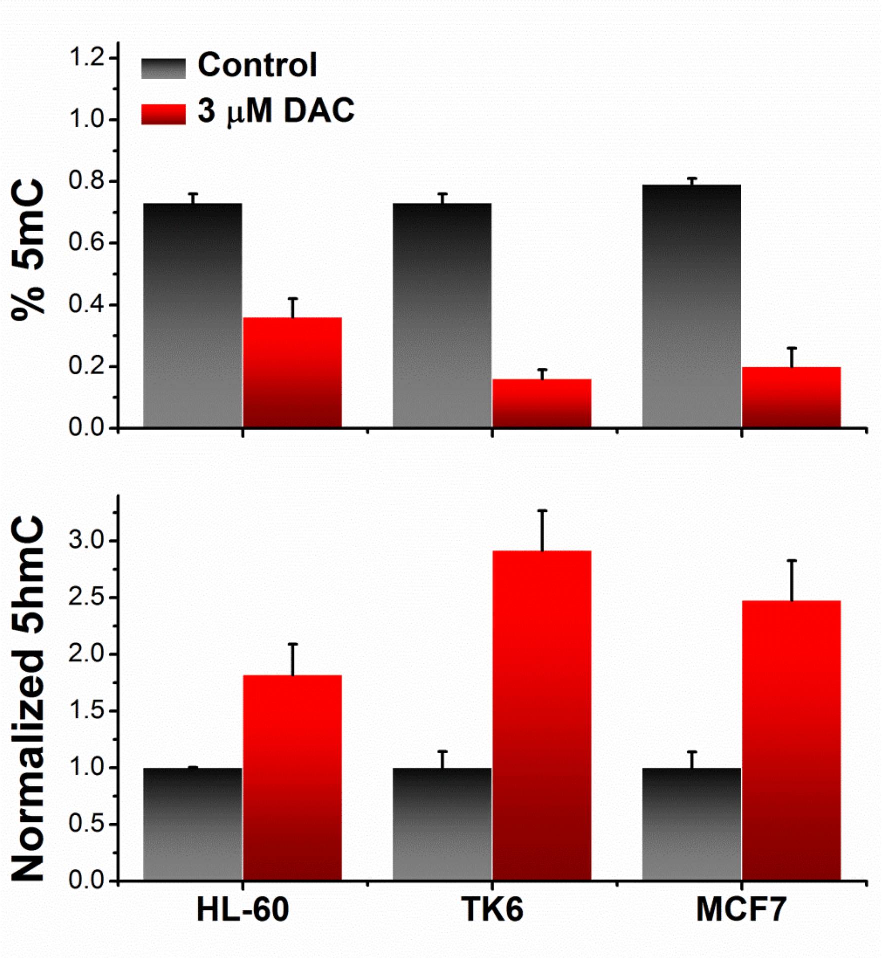

Supplementary Figure 5 *Changes of 5mC and 5hmC upon 3 μM DAC treatment in HL-60, TK6 and MCF7 cells.*

Analyte	Linear Range (fmol)	R^2	LOD (fmol)
**dC**	1.92 - 192000	0.999	0.36
**5mdC**	1.76 - 17600	0.999	0.09
**5hmdC**	1.65 - 16500	0.999	0.11

Supplementary Table 1 *Range and Limits of Detection of 5mdC and 5hmdC by LC-MS/MS. The indicated Linear Range, Coefficient Value (R2) and Limit of Detection (LOD) of LC-MS/MS were quantitated using calibration curves generated from authentic standards.*

Gene	Forward Strand Sequence (5'->3')	Reverse Strand Sequence (5'->3')
GAPDH	CAGCCTCAAGATCATCAGCA	TGTGGTCATGAGTCCTTCCA
DNMT1	TACCTGACGACCCTGACCTC	RCGTTGGCATCAAAGATGGACA
DNMT3A	TATTGATGAGCGCACAAGAGAGC	GGGTGTTCCAGGGTAACATTGAG
DNMT3B	GGCAAGTTCTCCGAGGTCTCTG	TGGTACATGGCTTTTCGATAGGA

Supplementary Table 2 *Primer Sequences used for RT-PCR*

Primer	Sequence (5’ -> 3’)
TET-CD (F)	atcgttGCTAGCAGCGGAAGTACACCCGCAATGGAACTGCCCACCTGCAGCTGTCTTGAT
TET-CD (R)	atcctaCCTGCAGGGACCCAATGGTTATAGGGCCCCGCAACGTG
EGFP (F)	atcctaCCTGCAGGGAAGCGGAAGTACACCCGCATGGTGAGCAAGGGCGAGG
EGFP (R)	tctacaaaGCGGCCGCCTACTTGTACAGCTCGTCCATG

Supplementary Table 3 *Primers used for molecular cloning. All primers listed are 5' to 3'. The restriction sites are represented in green, linkers in red, and the sites complementary to the inserts in black font color.*

SUPPLEMENTARY REFERENCES

1. Liu, C. C. *et al.* Global DNA methylation, DNMT1, and MBD2 in patients with systemic lupus erythematosus. *Lupus* **20**, 131-136, doi:10.1177/0961203310381517 (2011).

2. Jin, S.-G. *et al.* 5-Hydroxymethylcytosine Is Strongly Depleted in Human Cancers but Its Levels Do Not Correlate with IDH1 Mutations. *Cancer Research* **71**, 7360-7365, doi:10.1158/0008-5472.can-11-2023 (2011).

3. Chen, M.-L. *et al.* Quantification of 5-Methylcytosine and 5-Hydroxymethylcytosine in Genomic DNA from Hepatocellular Carcinoma Tissues by Capillary Hydrophilic-Interaction Liquid Chromatography/Quadrupole TOF Mass Spectrometry. *Clinical Chemistry* **59**, 824-832, doi:10.1373/clinchem.2012.193938 (2013).

4. McGovern, A. P., Powell, B. E. & Chevassut, T. J. T. A dynamic multi-compartmental model of DNA methylation with demonstrable predictive value in hematological malignancies. *Journal of Theoretical Biology* **310**, 14-20, doi:10.1016/j.jtbi.2012.06.018 (2012).

5. Sontag, L. B., Lorincz, M. C. & Georg Luebeck, E. Dynamics, stability and inheritance of somatic DNA methylation imprints. *J Theor Biol* **242**, 890-899, doi:10.1016/j.jtbi.2006.05.012 (2006).

6. Ito, S. *et al.* Tet proteins can convert 5-methylcytosine to 5-formylcytosine and 5-carboxylcytosine. *Science* **333**, 1300-1303, doi:10.1126/science.1210597 (2011).
